# *Id4* promotes cell proliferation in hepatocellular carcinoma

**DOI:** 10.1186/s40880-017-0186-7

**Published:** 2017-02-01

**Authors:** Yang Zhang, Li-Xing Zhang, Xiao-Qin Liu, Fang-Yu Zhao, Chao Ge, Tao-Yang Chen, Ming Yao, Jin-Jun Li

**Affiliations:** 1 Shanghai Medical College, Fudan University, Shanghai, 200032 P. R. China; 2 State Key Laboratory of Oncogenes and Related Genes, Shanghai Cancer Institute, Renji Hospital, Shanghai Jiaotong University School of Medicine, 25/Ln 2200, Xietu Road, Shanghai, 200032 P. R. China; 3 Qidong Liver Cancer Institute, Qidong, 226200 Jiangsu P. R. China

**Keywords:** Hepatocellular carcinoma, *Id4*, Proliferation

## Abstract

**Background:**

Hepatocellular carcinoma (HCC) is a common malignant tumor in the world, especially in China. As a member of the inhibitor of differentiation (*Id*) family, *Id4* has been reported to function in many cancer types, but relatively little is known about its role in HCC. The purpose of this study was to investigate the potential relationship between *Id4* and HCC development and the underlying mechanism involving the function of *Id4* in HCC.

**Methods:**

We used quantitative real-time polymerase chain reaction and Western blotting to examine the RNA and protein expression of *Id4*. In addition, we used Cell Counting Kit-8 assay and colony formation assay to identify the function of *Id4* in the regulation of cell proliferation in human HCC.

**Results:**

We found that the expression of Id4 protein was up-regulated in tumor tissues from HCC patients. Overexpression of *Id4* promoted HCC cell proliferation, clonogenicity in vitro, and tumorigenicity in vivo. *Id4* knockdown experiments showed that silencing *Id4* blocked the proliferation and colony formation ability of HCC cells in vitro. Furthermore, overexpression of CCAAT/enhancer-binding protein β inhibited *Id4* expression in HCC cells.

**Conclusion:**

*Id4* may be developed as a potent therapeutic agent for the treatment of HCC, but more details about the underlying mechanisms of action are needed.

## Background

Since the inhibitor of differentiation (*Id*) genes were first identified in 1990, more than 25 years of research has established that Id proteins are critical regulators in normal development and in cancer [1]. The *Id* family, a class of the helix-loop-helix (HLH) family, has four members; they lack a basic DNA-binding domain and function by forming heterodimers with other HLH family members to inhibit transcriptional activity [2]. Deregulation of Ids has been reported in many types of human cancers, such as prostate cancer, breast cancer, and ovarian cancer, and it may be strongly associated with poor prognosis and disease grade [3, 4].

Based on the sequence and structural property analyses, *Id4* is a remote homologue of other Ids despite sharing the conserved HLH domain, which suggests a potential novel role for *Id4* in the development of cancer [5]. Some data have shown that *Id1*, *Id2*, and *Id3* enhance proliferation and block differentiation in certain cell types, whereas *Id4* has an opposite function [6].

Worldwide and in China, hepatocellular carcinoma (HCC), which accounts for most (70%–90%) primary liver cancers, is a leading cause of cancer death [7]. As reported previously, Id proteins are involved in the development of HCC. Lee et al. [8] first reported the overexpression of *Id1* in HCC, which results in the inactivation of the p16^INK4a^/retinoblastoma pathway and leads to aberrant proliferation of HCC cells. Analysis of clinical samples showed a complex expression profile of *Id1*, *Id2*, and *Id3* and their relevance to the de-differentiation in HCC, which was different from the expression profile in breast, prostate, and colon carcinogenesis [9].

Recent studies have also demonstrated the function of *Id4* in breast and prostate cancers. Beger et al. [10] found that, in breast cancer, *Id4* played a role in the down-regulation of breast cancer 1 (*BRCA1*); its overexpression enhanced the tumorigenic potential of cells [11]. Moreover, Junankar et al. [12] found that *Id4* could be a vital regulator of mammary stem cells, because it could suppress the factors that contributed to luminal differentiation, maintaining the mammary stem cell pool; these results are consistent with the earlier finding demonstrating the positive role of Ids in cell growth but negative role in cell differentiation [10]. These findings show that *Id4* is a tumor promoter in breast cancer but that its function varies depending on the subtype and the developmental stage of cancer. On the contrary, other studies showed that *Id4* has a tumor-suppressive effect. In prostate cancer, *Id4* was down-regulated because of promoter hypermethylation, which provided evidence that *Id4* may be a tumor suppressor [13, 14]. However, the expression pattern and function of Id4 protein in HCC have not yet been determined.

In this study, we explored the potential relationship between *Id4* and HCC development as well as the underlying mechanism involving the function of *Id4* in HCC.

## Methods

### Cell lines and cell culture

The human HCC cell line SMMC-7721 was obtained from the Cell Bank of the Institute of Biochemistry and Cell Biology of the Chinese Academy of Sciences (Shanghai, China). The PLC/PRF/5, HepG2, SK-HEP-1, and Hep3B2.1-7 cell lines were purchased from the American Type Culture Collection (Manassas, VA, USA). The Huh7 cell line was obtained from the Riken Cell Bank (Tsukuba, Japan). The MHCC-97L and MHCC-LM3 cell lines were kindly provided by the Liver Cancer Institute, Zhongshan Hospital of Fudan University (Shanghai, China). All cell lines were maintained in Dulbecco’s modified Eagle’s medium (DMEM) (Sigma-Aldrich, St. Louis, MO, USA) supplemented with 10% fetal bovine serum (Hyclone, Logan, UT, USA) at 37 °C in a 5% CO_2_ incubator. Standard transient transfections for all cell lines were conducted using Lipofectamine 2000 (Life Technologies, Carlsbad, CA, USA) according to the manufacturer’s instructions.

### RNA extraction and quantitative real-time polymerase chain reaction

Total RNA was extracted from tissues and cells using TRIzol reagents (Invitrogen, Carlsbad, CA, USA). One microgram of total RNA was reversely transcribed with the Prime Script RT Reagent Kit (Perfect Real Time) (TaKaRa Biotechnology, Dalian, China). Polymerase chain reaction (PCR) analysis was performed using specific primers for the *Id4* gene: forward, 5′-GTGCGATATGAACGACTGCT-3′, and reverse, 5′-CAGGATCTCCACTTTGCTGA-3′. The expression levels were normalized using human *GAPDH* (glyceraldehyde-3-phosphate dehydrogenase) as an internal control: forward, 5′-AGAAGGCTGGGGCTCATTTG-3′, and reverse, 5′-AGGGGCCATCCACAGTCTTC-3′.

### Protein isolation and Western blotting

After specific treatments, proteins (20 µg) were separated using 12% SDS-PAGE (sodium dodecyl sulfate–polyacrylamide gel electrophoresis) and transferred to nitrocellulose membrane by an electroblotting Bradford assay, according to the manufacturer’s instructions (Sigma-Aldrich). The anti-Id4 monoclonal antibody (sc-365656, 1:100) and anti-C/EBPβ (CCAAT/enhancer-binding protein β) polyclonal antibody (sc-150, 1:400) were purchased from Santa Cruz Biotechnology (Santa Cruz, CA, USA), and the β-actin antibody (A3854, 1:10,000) was purchased from Sigma-Aldrich.

### Patient samples

Twenty-seven human HCC tissue samples were obtained from the Qidong Liver Cancer Institute (Qidong, Jiangsu, China). Tumor tissues and adjacent non-tumor tissues were used to detect the *Id4* mRNA and protein levels by real-time PCR and Western blotting. All procedures were performed under consensus agreements and in accordance with the China Ethical Review Committee.

### Immunohistochemical analysis

Fifty-seven human HCC tissue specimens were collected from patients who underwent surgical treatment at Qidong Liver Cancer Institute or at the First Affiliated Hospital of Zhejiang University (Hangzhou, Zhejiang, China). The 57 HCC patients, including 52 males and 5 females (mean age 45.0 years, ranging from 21.0 to 70.0 years), were followed up from November 21, 2001 to November 3, 2010. No patient received preoperative chemotherapy or radiotherapy. Informed consent was obtained from all patients, and the study was approved by the Ethics Committee of Fudan University.

Anti-Id4 polyclonal antibody (sc-491) was purchased from Santa Cruz Biotechnology. Immunohistochemical (IHC) analysis and signal evaluation were performed according to our previously described procedures [15]. All the HCC tissue slides were observed and photographed using an Axioskop 2 microscope (Carl Zeiss, Oberkochen, Germany). The IHC results were determined according to both staining intensity and the percentage of positive cells as described previously [15].

### Plasmid constructs for overexpression of *Id4* and *C/EBPβ* and RNA interference of *Id4*

The full-length human *Id4* and *C/EBPβ* open reading frame (ORF) were respectively generated and cloned into the lentiviral vector pWPLX (Addgene, Cambridge, MA, USA) at the *Bam*HI and *Eco*RI sites. The primers of *Id4* ORF used for cloning and testing were as follows. Forward: 5′-GGATCCATGAAGGCGGTGAGCCCG-3′; reverse: 5′- GAATTCTCAGCGGCACAGAATGCT-3′. The primers of *C/EBPβ (LAP1)* ORF used for cloning and testing were as follows. Forward: 5′-CGCGGATCCATGCAACGCCTGGTGGCCT-3′; reverse: 5′-CCGGAATTCCTAGCAGTGGCCGGAGGAG-3′. We ordered two small-interfering RNAs (siRNAs) and two short-hairpin RNAs (shRNAs) targeting *Id4*, which were synthesized and constructed, respectively, by the GenePharma (Shanghai, China). The sh*Id4* and shNC sequence (si*Id4*-1, 5′-GCACGUUCAUAAACAUUCUTT-3′; si*Id4*-2, 5′-CCCAACAAGAAAGUCAGCATT-3′; and siNC, 5′-TTCTCCGAACGTGTCACGT-3′) were cloned into the lentiviral vector pLVTHM (Addgene, Cambridge, MA, USA) to construct pLVTHM-sh*Id4* and pLVTHM-shNC. To verify the effect of overexpression or gene silencing, real-time PCR and Western blotting were performed.

### Colony formation assays

For colony formation assays, 500 SMMC-7721, 2000 MHCC-97L, and 3000 Huh7 cells per well were seeded in 6-well plates and cultured at 37 °C for 10–14 days. Then, the cells were fixed in 10% formaldehyde for 20 min and stained for 30 min with Giemsa solution (Sigma-Aldrich). Each measurement was performed in triplicate, and the experiments were each conducted three times.

### Cell growth assay

Cell proliferation analyses were performed using a WST-8 Cell Counting Kit-8 (CCK-8, Ruian Biotech, Shanghai, China). Cells (800 for SMMC-7721, 2000 for MHCC-97L, and 1500 for Huh7) suspended in DMEM (100 μL) with 10% fetal bovine serum were seeded in 96-well plates and incubated. After 24 h, 10 μL of CCK-8 solution was added to each well, and the cultures were incubated at 37 °C for 2 h. Absorbance was measured at 450 nm for 7 days. The relative absorbance value was calculated, and the absorbance value measured on the first day was used as a control. Each measurement was performed in triplicate, and the experiments were each conducted three times.

### Tumor xenograft assay

Six- to eight-week-old male BALB/c (nu/nu) mice were housed and treated under specific pathogen-free conditions at the Experimental Animal Center of Shanghai Jiaotong University School of Medicine (Shanghai, China). They were randomly divided into groups (eight mice per group) and maintained under standard conditions according to institutional animal guidelines. SMMC-7721-Id4 cells and their pWPXL vector control (SMMC-7721-pWPXL) cells (2 × 10^6^ cells per mouse) were separately injected subcutaneously into the right flank of nude mice. After 5 weeks, the mice were euthanized, and the xenograft tumors were weighted.

### Statistical analysis

Data were analyzed using SPSS 13.0 software (IBM Corporation, New York, NY, USA). Results are presented as mean ± standard deviation and compared using Student’s *t* test. The overall survival was calculated from the 4th month after hepatectomy to the date of death or the last follow-up. Univariate survival analysis was performed according to the Kaplan–Meier method, and differences in survival curves were assessed with the log-rank test. *P* values less than 0.05 were considered statistically significant.

## Results

### *Id4* expression in HCC samples and cell lines

**Fig. 1:**
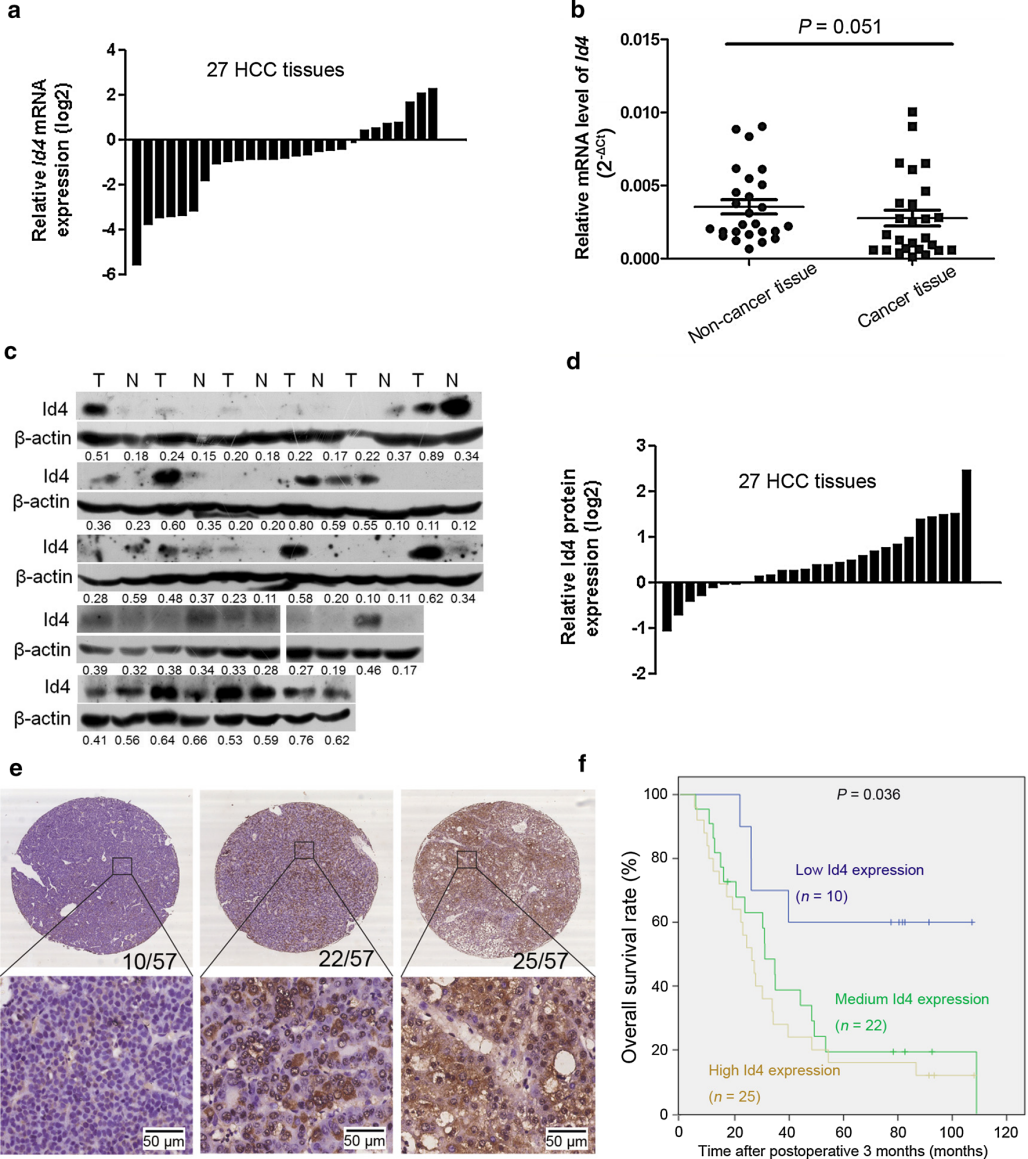
Inhibitor of differentiation 4 (*Id4*) was frequently up-regulated in hepatocellular carcinoma (HCC) cancer tissues at the protein level but not at the mRNA level. **a** The relative *Id4* mRNA expression in HCC tumor tissues is compared with the adjacent non-cancer tissues. **b** Compared with the adjacent non-cancer tissues, the mRNA expression of *Id4* is not significantly up-regulated in HCC tumor tissues. Paired Student’s *t* test, *P* = 0.051. *ns* not significant. **c** The up-regulated protein expression of Id4 is observed in 63.0% (17/27) of tumor tissues. Densitometry of Western blotting (measured by Image J) showed the relative protein expression (the numbers, IntDen_Id4_/IntDen_Actin_). **d** The relative Id4 protein expression in HCC tissues is compared with that in the adjacent non-cancer tissues. Densitometry of Western blotting (measured by Image J) showed the relative protein expression (IntDen_Id4_/IntDen_Actin_). **e** Representative Id4 immunostaining in a HCC tissue array containing 57 samples: no positive immunostaining is detected in 10 cases; Id4-positive cells are scattered or focally clustered in 22 cases; and diffuse staining pattern is present in 25 cases. The number of cases in each subset is shown in the right corner of the upper panel. Original magnifications: up ×4 and down ×40. T: tumor tissues; *N* adjacent non-cancer tissues. **f** Kaplan–Meier survival curve showing the overall survival of 57 HCC patients according to the Id4 expression level. *P < 0.05

In 27 pairs of human HCC specimens, we detected the expression of *Id4* by real-time PCR and Western blotting. Although *Id4* mRNA expression was up-regulated in adjacent non-cancer tissues as compared with cancer tissues from 27 cases (Fig. 1a), no significant difference was found between the two groups of tissues (Fig. 1b). However, 63.0% (17/27) of tumor samples showed up-regulated expression of Id4 protein; 18.5% (5/27) showed no difference; and the remaining 18.5% (5/27) showed a decrease of Id4 expression, compared with the corresponding non-cancerous liver samples (Fig. 1c, d). This may be partially due to the genetic heterogeneity in patients or contamination of tumor cells in some analyzed adjunctive liver tissues. Additionally, the Pearson correlation analysis results showed that protein levels of Id4 did not correlate with mRNA levels in tumor (*r* = −0.108, *P* = 0.592) or non-tumor (*r* = −0.010, *P* = 0.960) tissues.

To characterize the Id4 expression patterns, immunohistologic analysis was performed in a tissue array. Of the 57 HCC samples, Id4 expression was detected in 47 (82.5%) cases but not detected in 10 (17.5%) cases (Fig. 1e). In the 47 Id4-positive HCC samples, Id4-positive cells were scattered or focally clustered in 22 (38.6%) cases, and a diffuse staining pattern was present in 25 (43.9%) cases, which indicated that Id4 protein was overexpressed in most HCC tissues. To evaluate the association between the expression levels of Id4 and patient survival, a univariate analysis of overall survival was performed using the Kaplan–Meier method, and a log-rank test was performed. The result showed that patients with low Id4 expression had a higher survival rate when compared with medium or high Id4 expression groups (*P* = 0.036) (Fig. 1f). As an important transcriptional regulator [16], the increased level of Id4 expression may provide a clue regarding the role of Id4 in the development of liver cancer.

**Fig. 2:**
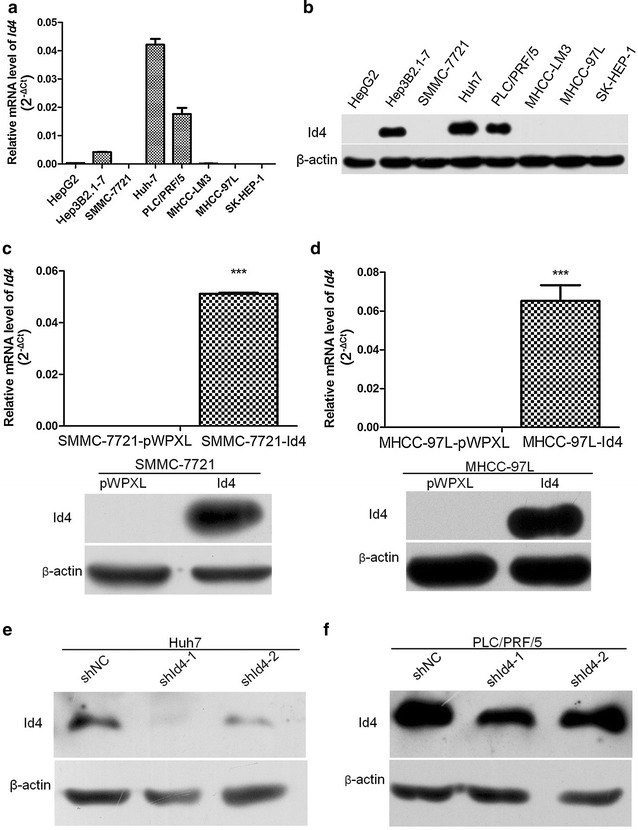
Expression of *Id4* in HCC cell lines. **a**, **b** The mRNA and protein levels of *Id4* in eight HCC cell lines. **c**, **d** Compared with the empty vector group, transfection with pMPXL-*Id4* markedly up-regulates the expression of *Id4* in SMMC-7721 and MHCC-97L. **e**, **f** In Huh7 and PLC/PRF/5 cells, stable transfection with sh*Id4* markedly down-regulates the expression of *Id4*. ***P < 0.001

We also analyzed the expression of *Id4* in HCC cell lines by real-time PCR and Western blotting (Fig. 2a, b). Id4 protein expression was markedly high in three cell lines (especially Huh7), and the mRNA level of *Id4* was in line with the protein expression. A positive correlation was found between *Id4* mRNA and protein expression in HCC cell lines (*r* = 0.799, *P* = 0.017), which was different from the expression determined in patient tissue samples.

### *Id4* stimulated proliferation in vitro

Two cell lines with low *Id4* expression, SMMC-7721 and MHCC-97L, were chosen for the construction of cell lines (SMMC-7721-Id4 and MHCC-97L-Id4) with overexpression of the *Id4* gene. Then, total RNA and proteins were collected, and the steady state of *Id4* was verified by real-time PCR and Western blotting (Fig. 2c, d). Two siRNA sequences were designed and synthesized, and endogenous *Id4* expression was silenced in Huh7 and PLC/PRF/5 cells. Using Western blotting, we confirmed the transfection and Id4 knockdown efficiency in Huh7 and PLC/PRF/5 cells (Fig. 2e, f).

**Fig. 3:**
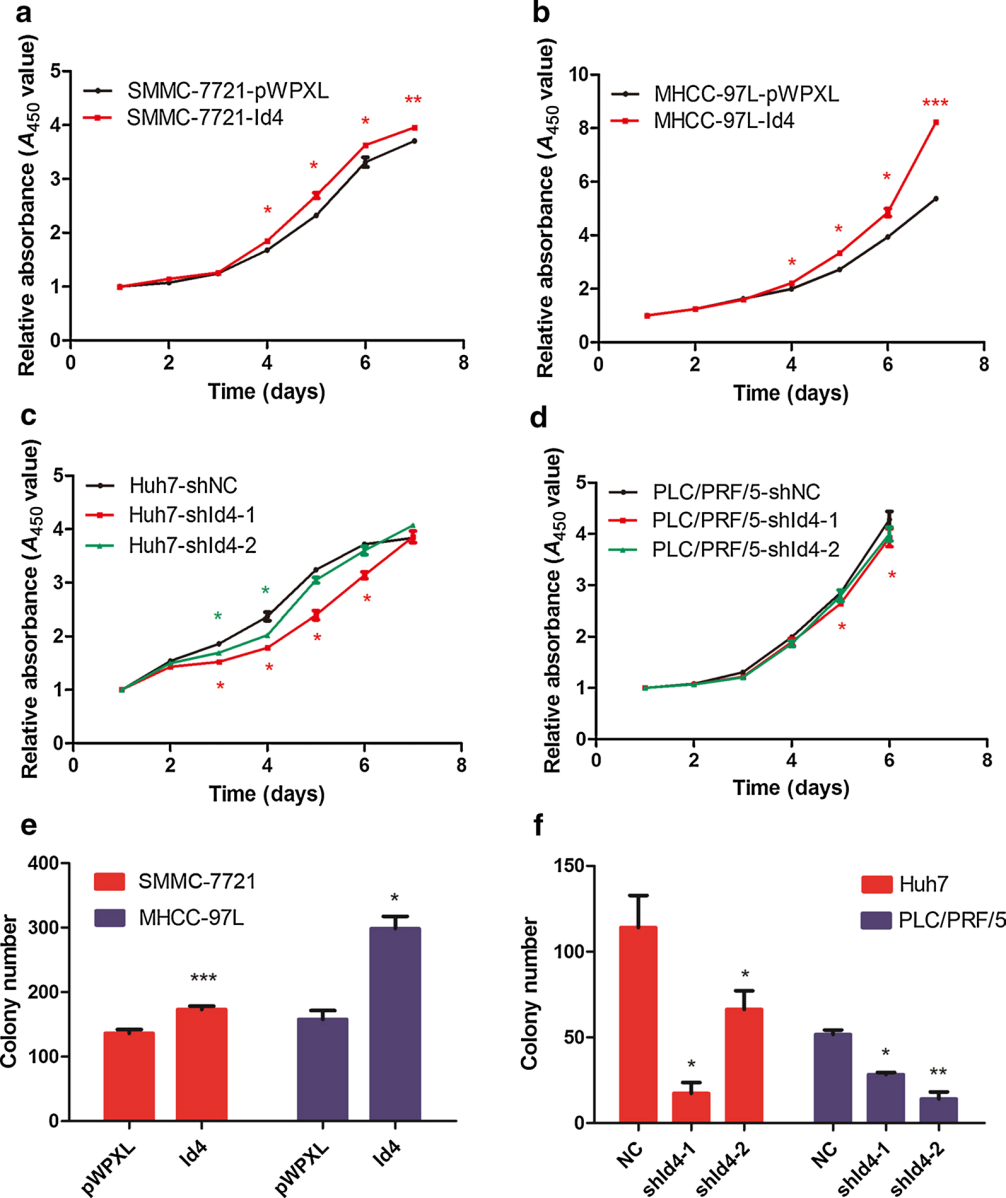
*Id4* promoted proliferation and colony formation of HCC cells in vitro. The Cell Counting Kit-8 (CCK-8) assay was performed to examine cell proliferation. **a**, **b** Up-regulation of *Id4* significantly promotes proliferation of SMMC-7721 and MHCC-97L cells. **c**, **d** *Id4* knockdown significantly suppresses cell proliferation in Huh7 and PLC/PRF/5. **e** The number of colonies increased significantly in SMMC-7721-Id4 and MHCC-97L-Id4 groups. **f** The numbers of colonies decreased remarkably after Id4 silence in Huh7 and PLC/PRF/5groups. *P < 0.05, **P < 0.01, ***P < 0.001

To assess the effect of *Id4* overexpression on HCC cell growth, we performed CCK-8 assays and colony formation assays. From the fourth day to the seventh day after culturing, significant differences (*P* = 0.046, 0.04, 0.041, and 0.009) were observed in proliferation between the SMMC-7721-pWPXL and the SMMC-7721-Id4 groups (Fig. 3a). A similar result (*P* = 0.040, 0.025, 0.015, and < 0.001) was obtained in MHCC-97L cells (Fig. 3b). A significant decrease in proliferation was observed in the Huh7-shId4-1 (*P* = 0.015, 0.022, 0.018, and 0.019), Huh7-shId4-2 (*P* = 0.019 and 0.024), and PLC/PRF/5-shId4-1 (*P* = 0.011 and 0.024) cells when compared with the control cells transfected with shNC (Fig. 3c, d). Furthermore, HCC cells with *Id4* overexpression formed more clones than the control cells transfected with empty vector pWPXL (SMMC-7721, *P* < 0.001; MHCC-97L, *P* = 0.028) (Fig. 3e). Moreover, when compared with the control cells transfected with shNC, the cells with down-regulated expression of *Id4* (transfected with shId4-1 and shId4-2) formed significantly less number of cell clones in Huh7 (*P* = 0.023 and 0.029) and PLC/PRF/5 (*P* = 0.012 and 0.002) cells (Fig. 3f), which suggested that *Id4* knockdown inhibited the cell growth ability of HCC cells. These results showed that increased accumulation of *Id4* contributed to proliferation and clonogenicity in SMMC-7721 and MHCC-97L cells.

### *C/EBPβ* overexpression inhibited *Id4* expression in Huh7 cells

**Fig. 4:**
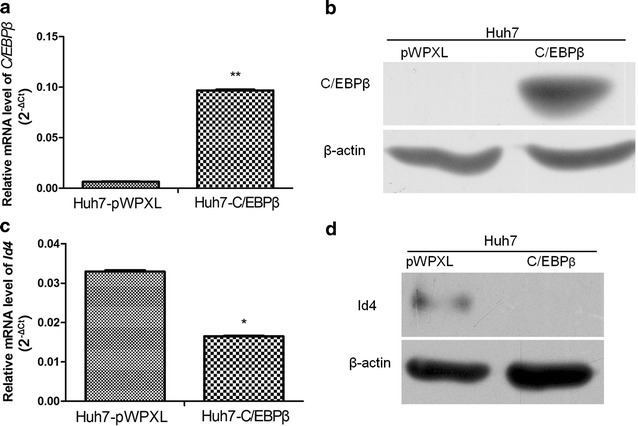
CCAAT/enhancer-binding protein β (*C/EBPβ*) overexpression inhibited *Id4* expression in Huh7 cells. **a**, **b** Real-time PCR and Western blotting analysis show that *C/EBPβ* mRNA and protein expression are significantly induced in Huh7 cells. **c**, **d** *Id4* mRNA and protein expression are inhibited in the *C/EBPβ*-overexpressing Huh7 cells. *P < 0.05, **P < 0.01

To investigate the mechanism that regulates *Id4* expression in HCC cells, we analyzed the *Id4* promoter region using bioinformatics methods. Bioinformatics analysis showed that there were *C/EBPβ*-binding sites in the *Id4* gene promoter region (Table 1). In Huh7 cells transfected with pWPXL-*C/EBPβ*, real-time PCR and Western blotting analysis showed that C/EBPβ mRNA and protein expression were significantly induced (Fig. 4a, b). Furthermore, *Id4* mRNA and protein expression were inhibited in the *C/EBPβ*-overexpressing Huh7 cells, which indicated that *C/EBPβ* may regulate *Id4* expression in HCC cells in a direct or indirect manner (Fig. 4c, d).

**Table 1:**
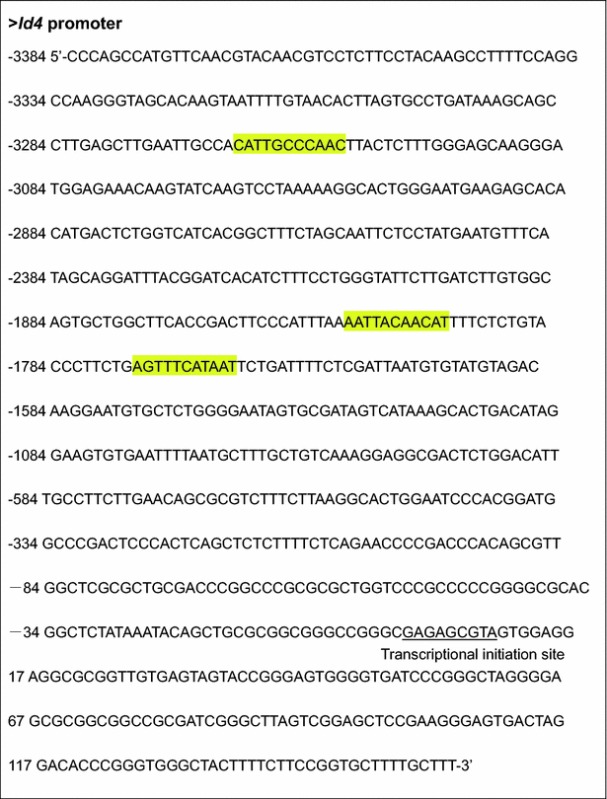
Predicted C/EBPß-binding sites in the *Id4* promoter sequence

Predicted binding sites of C/EBPß protein in the *Id4* promoter sequence are highlighted in yellow. The JASPAR database (http://jaspar.genereg.net/) was used in the bioinformatics analysis of DNA sequence

### *Id4* enhanced tumorigenicity potential in vivo

**Fig. 5:**
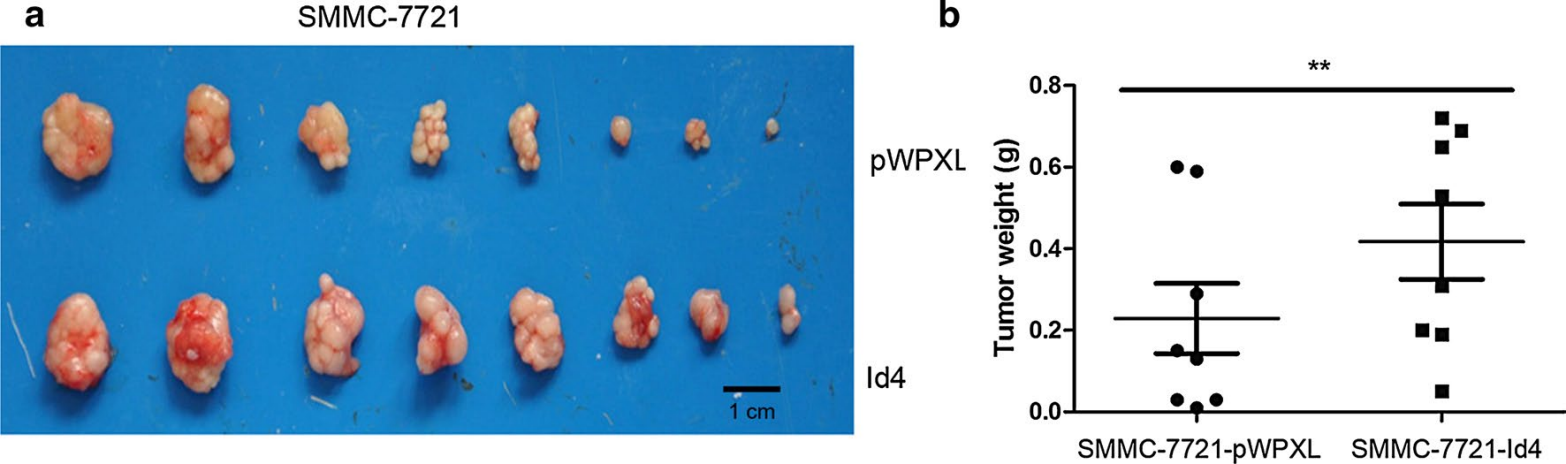
Overexpression of *Id4* enhanced the tumorigenic potential of HCC cells in vivo. SMMC-7721 cells that stably expressed *Id4* were injected subcutaneously into nude mice; the cells transfected with pWPXL vector were used as a control. After 5 weeks, the mice were euthanized, and the tumors were removed. **a** The xenograft tumors formed in nude mice. **b** The wet weight of the tumors. **P < 0.01

To determine whether overexpression of *Id4* has an effect on tumor growth in vivo, animal experiments was carried out. The result showed that Xenograft tumors in the SMMC-7721-pWPXL group were smaller than that in the SMMC-7721-Id4 group (Fig. 5). A significant difference in xenograft tumor weight was observed between the two groups (0.23 ± 0.24 g in the SMMC-7721-pWPXL group and 0.42 ± 0.26 g in the SMMC-7721-Id4 group, *P* = 0.003). Our results indicate that *Id4* overexpression significantly promoted the tumorigenicity of SMMC-7721 cells in this model system.

## Discussion

In this study, we found high expression of Id4 in three cell lines (Huh7, PLC/PRF/5, and Hep3B2.1-7), which was similar to our previous findings of the expression trend of cluster of differentiation 133 (CD133) protein in HCC [17]. We also found that the expression of Id4 protein was up-regulated in HCC patient tumor samples. Additionally, immunohistochemical analysis showed that Id4 protein was overexpressed in most HCC patient samples, which provides new insights into the role of Id proteins in human HCC [9]. Although a positive correlation was observed between the *Id4* mRNA and protein expression level in HCC cell lines, *Id4* mRNA and protein expression showed no significant correlation in the HCC patient samples. We speculate that this may be partially due to the complex cell types comprising the tumor tissues. Alternatively, the discrepancy between the *Id4* mRNA levels and protein levels may be a result of mediating activity by miRNAs or the different half-lives of proteins [18, 19]; either way, the detailed mechanism needs to be verified in further investigations. These findings indicate that *Id4* might be a potent therapeutic agent in HCC.

Previous findings show that, in cancer, there are many modes of activation of *Id* genes, such as transcriptional induction by oncoproteins or growth factor-directed signals, which provide convincing support for the theory that, in some contexts, *Id* genes function as oncogenes [20]. Moreover, many studies have suggested that Id proteins are involved in the cell cycle by interacting with cyclin D1, p21, or other molecules to promote progression through the S phase [21, 22]. An increased level of *Id4* has been observed in basal-like breast cancer, triple-negative breast cancer [23], glioblastoma [24], primary serous ovarian cancer [25], and melanoma [26], leading to the qualification of *Id4* as a proto-oncogene. The positive function of Id4 in tumor aggressiveness is best understood in breast cancer [21], but very little evidence is available regarding the role of Id4 protein in HCC.

To explore the role of the *Id4* gene in HCC, we used CCK-8 assays, colony formation assays, and the animal model, and found that sustained *Id4* overexpression was sufficient to increase HCC cell growth, enhance colony formation ability, and contribute to tumorigenesis. On the other hand, the *Id4* knockdown experiment showed the opposite results that silencing *Id4* blocked the proliferation and colony formation ability of HCC cells. Therefore, it is reasonable to conclude that *Id4* might function as a proto-oncogene in HCC.

*C/EBPβ* is an important transcriptional factor that has been implicated in many biological activities, including cancer progression. Loss of *C/EBPβ* regulation in breast cancer promoted the disease’s malignant progression by inducing epithelial-mesenchymal transition [27]. Our previous study showed that *C/EBPβ* could repress HCC cell migration and invasion by directly binding to the orosomucoid 2 (*ORM2*) promoter and inducing *ORM2* overexpression [28]. Many other studies have shown that *C/EBPβ* regulates transcriptional repression [29, 30]. For example, in the presence of a pro-inflammatory stimulus, *C/EBPβ* overexpression led to a decrease in *CD200R1* expression in microglial cells [30]. In our study, we found that *Id4* expression was inhibited in the *C/EBPβ*-overexpressing Huh7 cells. Therefore, we speculate that *C/EBPβ* may regulate *Id4* expression directly or indirectly in HCC cells; however, this needs to be verified by further investigations.

Overwhelming evidence suggests that Id proteins can be master regulators of cancer stem cells. The previous study showed that *Id1*/*Id3* regulates colon cancer-initiating cells (CC-ICs) by p21, and that, in colon cancer, *Id1*/*Id3* protects the tumor-initiating ability of CC-ICs from oxaliplatin [31]. Furthermore, in glioma, Jeon et al. [32] found a new *Id4*-*miR*-*9*-*SOX2* regulatory pathway that could affect the self-renewal activity of glioma cells, the chemoresistance of glioma stem cell (GSCs), and the maintenance of the stemness of induced GSCs. In our previous studies, we showed that *Ikaros* suppresses *CD133* expression and that *Id4* is one of the differentially expressed genes between the control and the *Ikaros*-overexpressing groups [17]. With reference to the expression of Id4 and CD133 and the function of *CD133* in HCC [33], we speculate that the *Id4* gene has a similar effect of *CD133* in HCC, but a great deal of work is needed to address this hypothesis.

In conclusion, we showed an increase in Id4 protein expression in HCC tissues and a promoting effect of *Id4* on proliferation in HCC cell lines in vitro and in vivo. Further investigation of the mechanisms and clinical value is greatly needed.
